# Assessment of central haemomodynamics from a brachial cuff in a community setting

**DOI:** 10.1186/1471-2261-12-48

**Published:** 2012-06-26

**Authors:** David Nunan, Siegfried Wassertheurer, Daniel Lasserson, Bernhard Hametner, Susannah Fleming, Alison Ward, Carl Heneghan

**Affiliations:** 1 Department of Primary Care Health Sciences, University of Oxford, Hythe Bridge St, Oxford, UK; 2 AIT Austrian Institute of Technology GmbH, Donau-City-Straße 1, 1220, Vienna, Austria

**Keywords:** Central pressure, Augmentation, Hypertension, Primary care, Validation

## Abstract

**Background:**

Large artery stiffening and wave reflections are independent predictors of adverse events. To date, their assessment has been limited to specialised techniques and settings. A new, more practical method allowing assessment of central blood pressure from waveforms recorded using a conventional automated oscillometric monitor has recently been validated in laboratory settings. However, the feasibility of this method in a community based setting has not been assessed.

**Methods:**

One-off peripheral and central haemodynamic (systolic and diastolic blood pressure (BP) and pulse pressure) and wave reflection parameters (augmentation pressure (AP) and index, AIx) were obtained from 1,903 volunteers in an Austrian community setting using a transfer-function like method (ARCSolver algorithm) and from waveforms recorded with a regular oscillometric cuff. We assessed these parameters for known differences and associations according to gender and age deciles from <30 years to >80 years in the whole population and a subset with a systolic BP < 140 mmHg.

**Results:**

We obtained 1,793 measures of peripheral and central BP, PP and augmentation parameters. Age and gender associations with central haemodynamic and augmentation parameters reflected those previously established from reference standard non-invasive techniques under specialised settings. Findings were the same for patients with a systolic BP below 140 mmHg (i.e. normotensive). Lower values for AIx in the current study are possibly due to differences in sampling rates, detection frequency and/or averaging procedures and to lower numbers of volunteers in younger age groups.

**Conclusion:**

A novel transfer-function like algorithm, using brachial cuff-based waveform recordings, provides robust and feasible estimates of central systolic pressure and augmentation in community-based settings.

## Background

Hypertension remains the most prevalent chronic disease in primary care, yet a considerable disease burden is attributable to inadequate blood pressure control [1]. As a result, research has aimed to better understand age related changes in arterial properties and alterations in underlying haemodynamic mechanisms contributing to blood pressure elevation [2-7]. Moreover, current modelling of cardiovascular risk relies on peripheral blood pressure measurements yet calibration between predicted risk and observed risk is not optimal [8]. Central measures of blood pressure (BP) and vascular function may improve risk prediction, potentially through more accurate diagnosis of hypertension, identification of vascular dysfunction not captured with peripheral measures or more accurate assessment of the pharmacodynamic responses to antihypertensive medication [9].

Augmentation of blood pressure is related to changes in central haemodynamics and pressure wave characteristics resulting from increased aortic wall stiffening and aortic remodelling [10-12]. The augmentation index is defined by a boost (augmentation) to blood pressure in late systole, attributed to the early return of wave reflection from peripheral sites [13]. It may have utility as a predictor of vascular events as large artery stiffening and wave reflections have been identified as the most important pathophysiological determinants of isolated systolic hypertension [14]. Regardless of the underlying mechanism, alterations in central and pulse wave characteristics are independently associated with increased risk of mortality and morbidity [15]. Therefore, pulse wave analysis is potentially a useful adjunct to cardiovascular risk assessment in clinical practice [16-19].

Despite the growing evidence base, pulse wave analysis assessment is yet to be adopted into clinical practice. This is most likely due to the lack of a pragmatic means in which to perform this analysis in real world settings. The evidence base to date has been provided from studies using non-invasive techniques (such as applanation tonomotry or mechanotransducers) that apply the current gold-standard methods for non-invasive pressure waveform analysis [16]. Indeed, reference data for augmentation index derived using applanation tonometry methods (SphygmoCor) demonstrate a non-linear increase with age and higher values for women compared with men [11]. However, these techniques require stringent assessment criteria and skilled operation that negates their application to settings outside of the artificial and selected research scenarios of previous studies.

To counter this, a number of devices offering more accessible arterial assessment have become available. These devices use an ordinary blood pressure cuff and automated blood pressure monitor with specialist software. The ARCSolver method (Austrian Institute of Technology, Vienna, Austria) provides estimates of central systolic and diastolic BP and augmentation index using a validated oscillometric device (Mobil-O-Graph NG 24 hour PWA: IEM, Germany) to record pressure waves and the application of a general transfer function. Under controlled laboratory settings the device demonstrates realistic and valid estimates of central BP and augmentation index and performance equals that of current reference non-invasive techniques (SphygmoCor) when compared with measures derived invasively [20],[21]. The ARCSolver method presents for the first time the realistic attainment of “central haemodynamic assessment for the masses” [17].

The aim of this study was therefore to perform an initial assessment of ARCSolver derived arterial and central haemodynamic parameters in an unselected general population away from the usual “laboratory” setting. Further validation of the device was sought by evaluating estimated parameters for the known age and gender related associations previously established for non-invasive reference methods [11].

## Methods

Participation in the study was voluntary and the study was approved by the Austrian Society for Clinical Pharmacology and Therapeutics and institute for hypertension patients.

### Study sample

We obtained estimates of central haemodynamics, including central systolic and diastolic BP and pulse pressure (PP), augmentation pressure (AP) and index (AIx) from 1,903 members of the general public during the period March – October 2007. Estimates were obtained in various public locations (e.g. shopping malls) in the city of Vienna, Austria. This formed part of the annual “Wiener Herz-Kreislauf (A Heart for Vienna) Event”, an annual initiative since 2003 for cardiovascular prevention and awareness building. Participants were approached and asked if they would be willing to have their blood pressure assessed using the ARCSolver method. Age was restricted to >14 years with no upper limit. Informed consent was obtained from all participants and parents where necessary (<16 years). Consent was given for the use of anonymised data for subsequent analysis and relevant dissemination. The study protocol adhered to the Declaration of Helsinki.

### Instrumentation

The ARCSolver method is commercially available in the oscillometric Mobil-O-Graph NG® 24-hour ambulatory BP and PWA monitor (IEM; Stolberg, Germany). The device is Food and Drug Administration and Conformité Européenne approved and its blood pressure detection unit is validated according to British Hypertension Society [22] and European Society of Hypertension [23] recommendations. The algorithm for the generation of central systolic BP and aortic blood pressure curves, using the oscillometric method, have been reported previously [20,24] but are briefly explained. After the conventional oscillometric BP assessment, peripheral pressure waves are recorded, using the brachial cuff, at diastolic BP level for ≈ 10 seconds. Following digitization, a 3-step algorithm is applied. First, the single pressure waves are verified for their plausibility by testing minima position and corresponding wavelengths. Minima are detected by means of an iterative procedure evaluating higher order time derivatives of the pressure signal. The second stage involves comparison of all single pressure waves with one another to recognize artefacts. Aortic pulse waves are then generated via a general transfer function. Modulus and phase characteristics of the ARCSolver transfer function are available [20]. Finally, the coherence of the measured parameters is verified and displayed within the Mobil-O-Graph NG software package which also allows visual inspection to unveil consistently recorded intrinsic waveform distortion manually. The entire process takes between 2 and 3 minutes.

### Measurement of blood pressure and pulse wave analysis

All recordings were performed in seclusion within a Red Cross “medics” tent by trained paramedics familiarised with the ARCSolver method and standard oscillometric BP measurement procedures. Trained staff instructed each volunteer to sit on a chair with legs uncrossed and feet flat on the floor, and their back resting against the chair backrest. An appropriately-sized blood pressure cuff was then attached to the volunteer’s left or right arm. The volunteers arm was then rested on a table placing the cuff at approximately heart level. Participants were given a short rest (~5 minutes) prior to commencement of BP recordings during which time resting heart rate (HR) was recorded. The Mobil-O-Graph first performed a brachial BP recording to determine systolic and diastolic BP. This was followed by a 10-second PWA recording with the cuff inflated at the diastolic BP level.

Mean arterial pressure (MAP) and pulse pressure (PP) were calculated from peripheral systolic and diastolic BP as MAP = diastolic BP + 0.4(systolic BP – diastolic BP) [25] and PP = systolic BP – diastolic BP. The ratio of peripheral to central PP was used to provide PP amplification. Finally, a cut-off of 120 mmHg was used to determine an erroneous peripheral diastolic BP reading. Measurements of pulse wave velocity from the ARCSolver method are undergoing validation against invasive and non-invasive reference standard techniques. For this reason, data are only presented for measures of central haemodynamics and augmentation for which validation data have been published [20,26].

### Statistical analysis

We performed descriptive analyses using PASW (formally SPSS) statistics software (Version 18) and present data graphically, analysed according to age-specific and gender groups. We compared data in the present study with published data obtained from non-invasive reference methods (e.g. SphygmoCor). We categorised the sample into age deciles (<30, 30-39, 40-49, 50-59, 60-69, 70-79, >79) and BP categories (optimal, <120/80; normal, >120/80 to <130/85; high normal, >130/85 to <140/90; Grade I hypertension, >140/90 to <160/100; and Grade II/III hypertension, >160/100 mmHg), accommodating those used in comparative studies [11],[25].

All parameter values in categories according to age and grade of hypertension are given as mean and standard deviation. We assessed differences between and within groups using analysis of variance (ANOVA) and independent *t*-tests respectively. Associations were assessed using Pearson’s correlation. Significance was set at an alpha of 0.05. We examined data for face validity with established age and gender associations in the whole sample dataset and separately in a sample of participants with a systolic BP <140 mmHg (i.e. normotensive) as in previous studies [11,25].

## Results

The average values for seated haemodynamic parameters in all volunteers (i.e. with a peripheral systolic BP above and below 140 mmHg) are presented in Table 1, grouped by gender and decade of age. Data from 110 participants were excluded due to missing or erroneous values with analysis therefore performed on data from 1,793 volunteers. The sample consisted of 739 (41 %) males with a mean age of 54 years (range 14 to 95) and 1,054 females with a mean age of 57 years (range 14 to 99).

**Table 1:** Participant characteristics and seated haemodynamic parameters by age for males (M) and females (F)

**Decile parameter**		**<30 yrs**	**30-39 yrs**	**40-49 yrs**	**50-59 yrs**	**60-69 yrs**	**70-79**	**>80**	**ANOVA**		
	**M**	**n = 51**	**n = 96**	**n = 138**	**n = 181**	**n = 151**	**n = 87**	**n = 35**	**Age**	**Gender**	Age*gender
	**F**	**n = 81**	**n = 108**	**n = 127**	**n = 193**	**n = 281**	**n = 161**	**n = 103**			
psystolic BP (mm Hg)	M	128 ± 14	127 ± 17	132 ± 18	136 ± 22	140 ± 21	145 ± 23	145 ± 27	<0.001	0.003	0.002
	F	116 ± 15	117 ± 18	128 ± 21	133 ± 19	141 ± 20	145 ± 21	147 ± 23			
pdiastolic BP (mm Hg)	M	72 ± 10	77 ± 11	80 ± 11	80 ± 13	81 ± 12	82 ± 14	76 ± 13	<0.001	<0.001	0.004
	F	67 ± 9	68 ± 12	76 ± 14	76 ± 12	79 ± 13	80 ± 12	78 ± 13			
pPP (mm Hg)	M	56 ± 12	50 ± 11	52 ± 12	56 ± 15	59 ± 14	63 ± 17	69 ± 20	<0.001	NS	0.016
	F	49 ± 10	49 ± 12	52 ± 12	57 ± 15	61 ± 14	65 ± 16	69 ± 15			
MAP (mm Hg)	M	90 ± 10	93 ± 17	97 ± 12	99 ± 15	101 ± 14	103 ± 16	99 ± 17	<0.001	<0.001	<0.001
	F	83 ± 10	84 ± 13	93 ± 16	95 ± 13	100 ± 14	101 ± 13	101 ± 15			
csystolic BP (mm Hg)	M	111 ± 12	114 ± 17	120 ± 17	124 ± 20	129 ± 21	134 ± 23	132 ± 27	<0.001	0.021	0.011
	F	102 ± 14	105 ± 17	116 ± 20	122 ± 18	130 ± 20	134 ± 20	135 ± 23			
cPP (mm Hg)	M	37 ± 8	35 ± 9	38 ± 11	42 ± 13	46 ± 13	50 ± 14	54 ± 18	<0.001	NS	NS
	F	33 ± 8	35 ± 10	38 ± 10	44 ± 12	48 ± 13	52 ± 14	55 ± 14			
PP Amp (ratio)	M	1.52 ± 0.17	1.42 ± 0.14	1.39 ± 0.15	1.35 ± 0.14	1.29 ± 0.12	1.28 ± 0.13	1.29 ± 0.14	<0.001	0.001	NS
	F	1.47 ± 0.16	1.40 ± 0.12	1.37 ± 0.12	1.32 ± 0.11	1.29 ± 0.11	1.26 ± 0.12	1.27 ± 0.12			
AP (mmHg)	M	6 ± 4	7 ± 5	9 ± 6	11 ± 7	14 ± 8	15 ± 8	16 ± 10	<0.001	<0.001	NS
	F	8 ± 5	10 ± 6	11 ± 5	15 ± 7	18 ± 9	20 ± 10	20 ± 9			
AIx (%)	M	14 ± 10	20 ± 10	23 ± 10	25 ± 10	29 ± 10	28 ± 10	28 ± 11	<0.001	<0.001	NS
	F	23 ± 11	28 ± 9	29 ± 8	33 ± 9	35 ± 10	36 ± 10	35 ± 11			
HR (beats/min)	M	74 ± 12	73 ± 12	73 ± 11	75 ± 11	77 ± 12	75 ± 13	77 ± 14	0.009	NS	NS
	F	75 ± 12	74 ± 11	75 ± 12	75 ± 11	77 ± 14	77 ± 12	76 ± 13			

Data are mean ± SD. ANOVA columns represent results from the two-way analysis of variance for age and gender including the interaction term.

AP = augmentation pressure; AIx = augmentation index; cPP = central pulse pressure; csystolic BP = central systolic blood pressure; HR = heart rate; MAP = mean arterial pressure; pdiastolic BP = peripheral diastolic blood pressure; PP Amp = pulse pressure amplitude; pPP = peripheral pulse pressure; psystolic BP = peripheral systolic blood pressure.

### Central haemodynamic parameters

**Figure 1:**
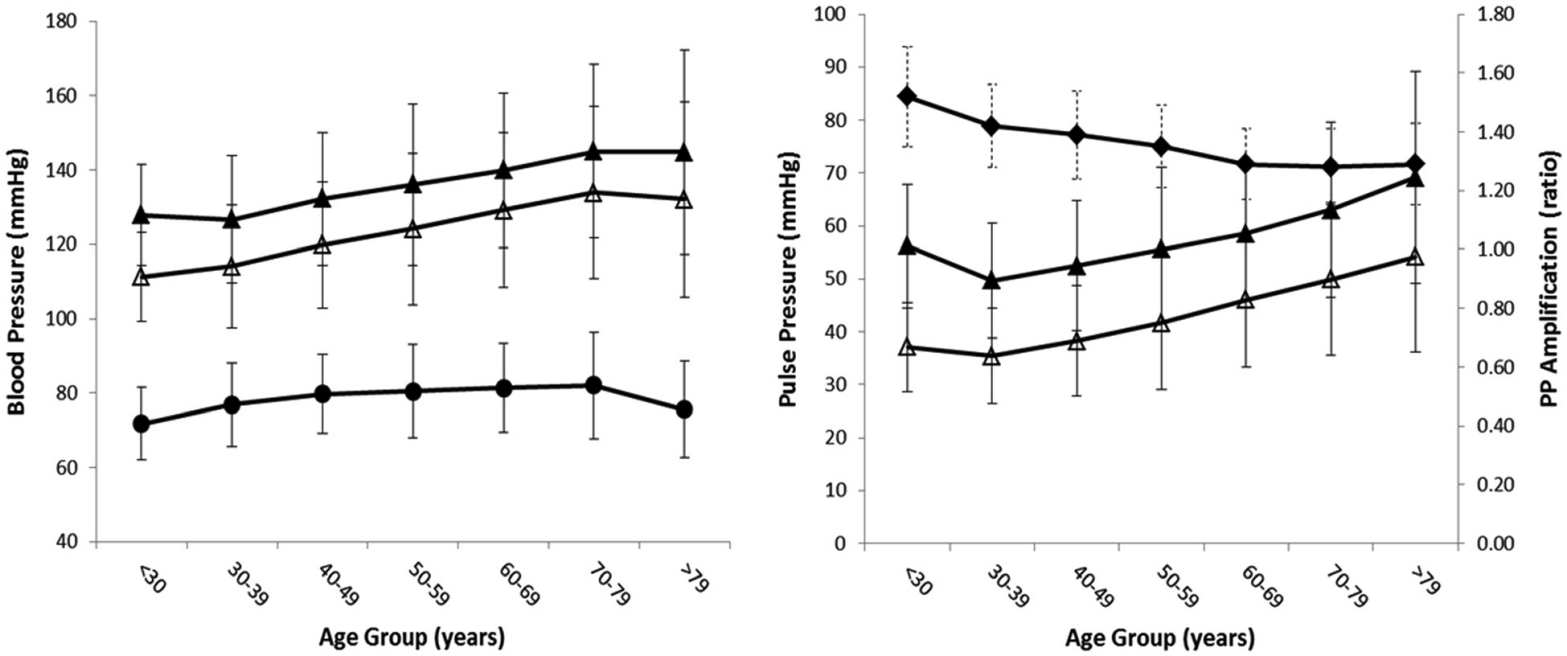
** Blood pressure (left panel) and pulse pressure and its amplification (right panel) averaged for deciles of age for males.** Symbols represent peripheral systolic (▴), diastolic (●) and central systolic (Δ) blood pressures (left panel); peripheral (▴) and central (Δ) pulse pressure and their amplification ratio (♦) (right panel).

**Figure 2:**
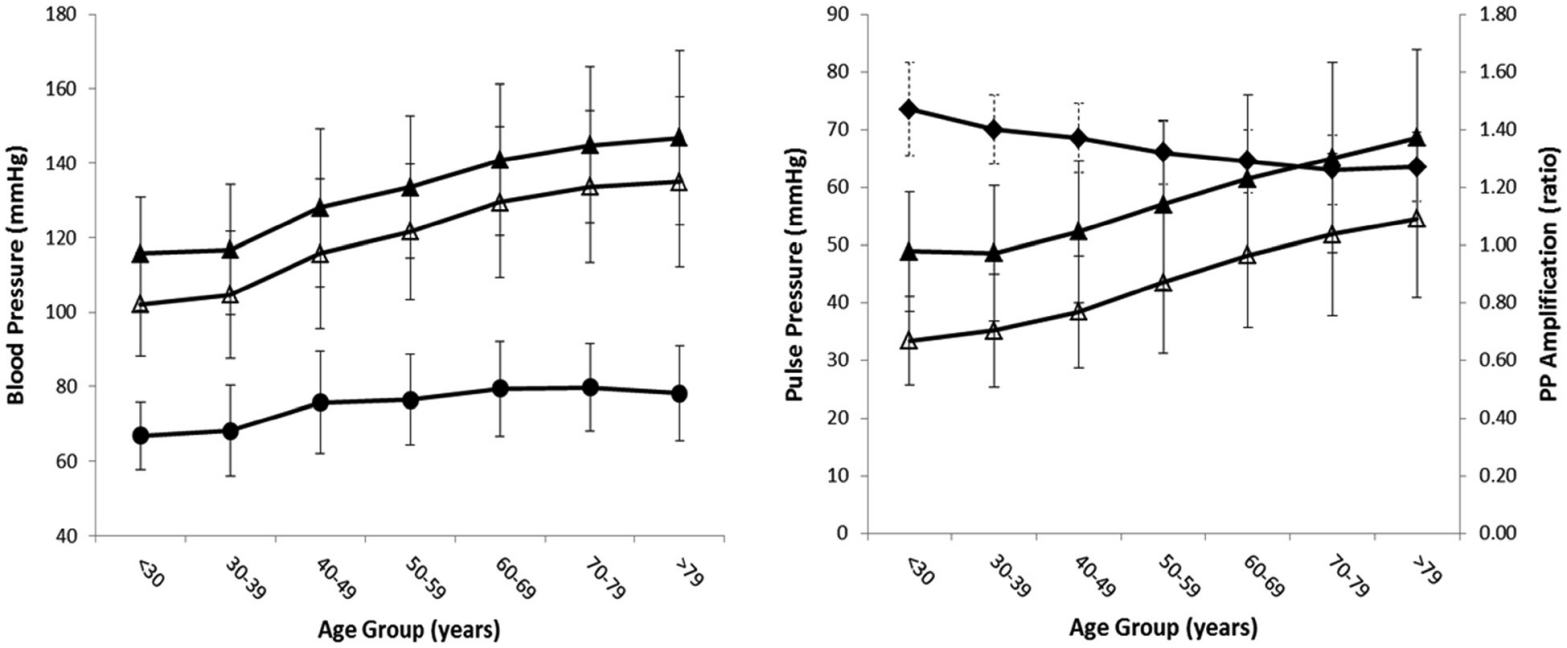
** Blood pressure (left panel) and pulse pressure and its amplification (right panel) averaged for deciles of age for females.** Symbols represent peripheral systolic (▴), diastolic (●) and central systolic (Δ) blood pressures (left panel); peripheral (▴) and central (Δ) pulse pressures and their amplification ratio (♦) (right panel).

Men and women demonstrated a similar increase in peripheral systolic blood pressure with age, whereas diastolic blood pressure increased to approximately 50 years and then plateaued. As a result, peripheral PP widened from 50 years onward with differences between men and women in the manner (p = 0.016, ANOVA gender by age) but not magnitude (p = 0.96, ANOVA gender) of change. Central systolic blood pressure demonstrated similar changes with age as those observed for peripheral systolic blood pressure (Figures 1 and 2). There were no differences between men and women in the manner or magnitude of change in central PP (p = 0.19 and 0.34, ANOVA, gender by age and gender respectively). The overall result was a non-linear decrease in PP amplification with age that was more marked in volunteers under 50 years (Figures 1 and 2). Within all age groups, PP amplification was significantly higher in males than in females (p < 0.001, ANOVA).

Performing the above analysis in volunteers with a peripheral systolic BP <140 (i.e. normotensive) revealed similar findings except increases with age were not as marked and women demonstrated a marginally greater increase in central PP compared with men (p = 0.03, ANOVA, Table 2).

**Table 2:** Participant characteristics and seated haemodynamic parameters by age for normotensive males (M) and females (F)

**Decile parameter**		**<30 yrs**	**30-39 yrs**	**40-49 yrs**	**50-59 yrs**	**60-69 yrs**	**70-79**	**>80**	**ANOVA**		
	**M**	**n = 42**	**n = 73**	**n = 100**	**n = 105**	**n = 77**	**n = 35**	**n = 18**	**Age**	**Gender**	Age*gender
	**F**	**n = 74**	**n = 95**	**n = 91**	**n = 128**	**n = 129**	**n = 64**	**n = 35**			
psystolic BP (mm Hg)	M	123 ± 10	119 ± 10	123 ± 10	121 ± 11	124 ± 12	124 ± 12	122 ± 14	<0.001	0.001	<0.001
	F	113 ± 12	111 ± 11	118 ± 12	123 ± 12	123 ± 11	124 ± 11	122 ± 12			
pdiastolic BP (mm Hg)	M	70 ± 9	73 ± 8	76 ± 8	74 ± 9	74 ± 9	72 ± 10	68 ± 10	<0.001	<0.001	0.01
	F	66 ± 8	65 ± 9	70 ± 10	73 ± 10	71 ± 10	72 ± 9	67 ± 9			
pPP (mm Hg)	M	53 ± 10	46 ± 8	47 ± 8	47 ± 8	49 ± 9	50 ± 8	53 ± 9	<0.001	NS	<0.001
	F	47 ± 9	46 ± 9	48 ± 8	50 ± 9	52 ± 10	52 ± 10	54 ± 9			
MAP (mm Hg)	M	88 ± 8	88 ± 8	91 ± 8	90 ± 9	91 ± 9	90 ± 10	86 ± 10	<0.001	<0.001	<0.001
	F	81 ± 9	81 ± 9	86 ± 10	89 ± 10	89 ± 9	90 ± 8	85 ± 9			
csystolic BP (mm Hg)	M	108 ± 9	107 ± 9	111 ± 9	110 ± 11	113 ± 12	113 ± 14	112 ± 15	<0.001	0.002	<0.001
	F	100 ± 11	100 ± 11	106 ± 12	112 ± 12	113 ± 11	114 ± 11	111 ± 11			
cPP (mm Hg)	M	35 ± 7	32 ± 7	34 ± 6	34 ± 7	38 ± 8	39 ± 8	41 ± 7	<0.001	0.03	0.006
	F	32 ± 6	33 ± 7	35 ± 7	38 ± 8	40 ± 8	40 ± 8	42 ± 8			
PP Amp (ratio)	M	1.52 ± 0.18	1.44 ± 0.14	1.39 ± 0.12	1.37 ± 0.13	1.32 ± 0.10	1.32 ± 0.15	1.31 ± 0.12	<0.001	0.004	NS
	F	1.48 ± 0.17	1.40 ± 0.12	1.38 ± 0.12	1.33 ± 0.11	1.31 ± 0.10	1.30 ± 0.11	1.30 ± 0.11			
AP (mmHg)	M	5 ± 4	6 ± 4	8 ± 4	8 ± 4	9 ± 4	10 ± 6	11 ± 5	<0.01	<0.001	NS
	F	8 ± 5	9 ± 4	10 ± 4	12 ± 5	12 ± 5	13 ± 6	14 ± 6			
AIx (%)	M	15 ± 10	19 ± 10	22 ± 10	23 ± 10	26 ± 8	26 ± 11	25 ± 11	<0.001	<0.001	NS
	F	23 ± 11	28 ± 9	29 ± 9	32 ± 9	33 ± 9	33 ± 10	33 ± 11			
HR (beats/min)	M	72 ± 10	72 ± 11	71 ± 10	75 ± 11	78 ± 12	76 ± 13	76 ± 14	0.03	NS	NS
	F	75 ± 13	74 ± 11	75 ± 13	75 ± 11	77 ± 14	76 ± 13	75 ± 14			

Data are mean ± SD. ANOVA columns represent results from the two-way analysis of variance for age and gender including the interaction term.

AP = augmentation pressure; AIx = augmentation index; cPP = central pulse pressure; csystolic BP = central systolic blood pressure; HR = heart rate; MAP = mean arterial pressure; pdiastolic BP = peripheral diastolic blood pressure; PP Amp = pulse pressure amplitude; pPP = peripheral pulse pressure; psystolic BP = peripheral systolic blood pressure.

### Augmentation parameters

**Figure 3:**
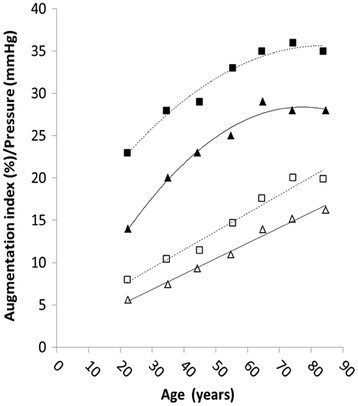
** Regression curves representing the effect of age on augmentation pressure and index.** Symbols represent augmentation pressure and augmentation index for males (▴ andΔ, solid line) and females (■ and □, dashed lines).

Both AP and AIx were significantly and positively correlated with age for both men (r = 0.37, p < 0.001) and women (0.35, p < 0.001), with values higher in women compared with men on average (RR 1.32; 95 % CI 1.18 to 1.48, p < 0.001) and in each decile of age (p < 0.001,ANOVA, Table 1). For both men and women the changes in AP with age were linear. Changes in AIx were non-linear, with a higher inter-decile increase in those under 50 years of age (Figure 3).

**Figure 4:**
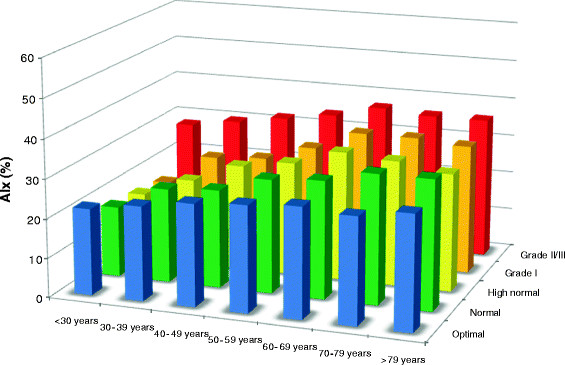
Observed values for augmentation index: mean value according to age and blood pressure (BP) category AIx, augmentation index.

Figure 4 shows there was a significant effect of blood pressure group on augmentation index (p < 0.001, ANOVA). Post hoc analysis revealed those with a Grade I hypertensive systolic BP had a significantly higher augmentation index compared to optimal (p < 0.001) and normal (p < 0.001) BP groups. Those with Grade II/III hypertension had a significantly higher augmentation index than volunteers in any other group (p < 0.001).

## Discussion

The aims of this study were to determine if estimates of central haemodynamics and augmentation parameters could demonstrate known associations with age and gender when obtained from an oscillometric device and if these measures were robust to a “real world” scenario. The results show that oscillometric derived estimates demonstrate the same characteristic age and gender associations observed in broadly similar populations using reference standard non-invasive techniques under controlled conditions. Combined with data that demonstrate accurate estimates from the device under laboratory conditions [20,21], these findings indicate that it is now feasible to derive estimates of central haemodynamics and augmentation index outside the laboratory setting and scenario.

The importance of central measures of BP and arterial stiffness stems from evidence of a strong prediction of adverse events in both general and diseased populations. A recent review of 11 longitudinal studies in 5,648 subjects identified a 9 % increase in the risk of a cardiovascular event for every 10 mmHg increase in central BP [15]. An increase of central pulse pressure by 10 mmHg was associated with a 14 % increase in the risk of a CV event. Finally, an increase in augmentation index of 10 % was associated with a 32 % increase in risk of a cardiovascular event and increased the risk of death from all causes by 38 % [15]. Additional analysis of five papers looked at the predictive power of central pressures versus that of peripheral pressures. Whilst no difference for systolic BP was observed, there was a trend for central PP to be more predictive than peripheral PP (p = 0.057) [15].

Data for central haemodynamic and augmentation parameters are presented in a format that allows direct comparison with those from the study of McEniery et al [11] and to our knowledge these are the first data to confirm their findings. In their 2005 study, the authors demonstrated a distinct non-linear association between age and augmentation index in a large cohort of normotensive adults [11]. The authors reported a greater age-related change in younger people (<50 years) compared with their older counterparts and suggest that augmentation index may provide a more sensitive marker of arterial aging in this age group. In over 50 year olds, a linear association across all age groups with central systolic BP differed to a marked increase in peripheral systolic BP.

We wanted to demonstrate whether data from a new brachial cuff-based method would mimic data obtained by McEniery [11] thus indicating the feasibility of wide scale clinical adoption of central BP and augmentation parameters. We observed non-linear age-related changes in augmentation index similar to those of McEniery [11]. In both men and women, augmentation index increased with age until approximately 50-60 years of age, after which a levelling off is seen. Moreover, females demonstrated higher values for augmentation index for each decade of age. Linear associations between age and central systolic BP and AP were also observed. Therefore age and gender associations with measures of central haemodynamics and augmentation parameters obtained using the commercially available oscillometric device mirror those observed for measures obtained using current reference standard techniques under more strict and controlled settings.

Similar outcomes were observed when analysis was performed in normotensive (systolic BP < 140 mmHg) participants only (Table 2) and indicates that age and gender associations observed for augmentation index are not restricted to individuals with a normal blood pressure but should also be observed in patients with a BP >140 mmHg. Combined with the recent presentation of data validating against invasive standard measures (catheter at the ascending aorta) [26], the oscillometric device affords estimates that are both robust and feasible in community based settings. We recommend that the Mobil-O-Graph device with ARCSolver method is ready for adoption into large cohort studies and trials for the assessment of central BP and augmentation parameters.

Our study is not without its limitations and many questions remain unanswered. Whilst we have demonstrated known age and gender associations for parameters derived from the oscillometric device, we have not demonstrated similar values for all parameters across all age groups compared with those from reference standard and other non-invasive techniques. This is particularly true for measures of augmentation index in younger participants (e.g. < 30 years of age). We were not able to assess statistically the nature of the systematic overestimation of augmentation index. Validation studies have demonstrated the ARCSolver method provides accurate estimates that are within recommended acceptable limits when compared to gold-standard invasive [21] and reference non-invasive [20,21] derived estimates. However, for measures of augmentation index, the standard deviation between ARCSolver and estimates from the SphygmoCor device was 7.9 % [20]. This suggests augmentation index estimated in the same person by the two devices may differ by as much as 31 %. Applying this value in the present study, observed values of between 6 % and 8 % higher in men and women aged 30 to 39 and 5 % and 4 % lower in men and women aged >79 years compared with published data [11] could be explained by device variation. These differences in AIx observed in comparison studies may be explained by differences in sampling rates (e.g. the cuff-based method has a resonant frequency around 60 Hz) and algorithms to determine inflection point of the pressure wave (cuff method uses the 4^th^ derivative rule, the algorithm for the SphygmoCor is not in the public domain). It is also likely that the two methods adopt differing averaging procedures. It is important to note, however, that the reference non-invasive methods themselves (e.g. SphygmoCor) display a degree of error when compared to invasive measures [27] and therefore neither method provides a “true” measurement of augmentation index (which can only be obtained via invasive catheterisation).

More pragmatic factors offer additional explanation for the observed discrepancies. The smaller sample size of the present study meant small group sizes in some cases (e.g. male, >79 years, BP <140 mmHg, n = 11). For this reason we decided not to include certain groups that were presented in the comparison studies. For example, McEniery et al [11] included separate groups for male and female subjects <20 years of age. In our study such grouping would have presented with only 10 and 18 eligible participants respectively. The grouping of all individuals under the age of 30 may reflect the higher values for augmentation index observed compared to previous data. This finding is emphasised by the fact that discrepancies are smaller between the directly comparable groups (e.g. 30 to 39, 40 to 49 etc) and lower values were observed when younger age groups (<20 years) were assessed (data not shown). A larger cohort of participants <20 and <30 years of age would allow for a better comparison of values for AIx and also the age associated change in central and peripheral BP for these age groups.

Comparison studies included only “healthy” subjects in their reference group, identified as those with no concomitant medical conditions (e.g. diabetes, heart disease, obesity) [11]. Whilst we could control for hypertension and other estimated parameters (e.g. systolic/diastolic BP, PP, MAP) we could not however control for participant characteristics in the same manner. The implications in terms of observed discrepancies are important particularly when factors such as height, weight, dyslipidaemia and smoking have been shown to significantly influence measures of central haemodynamics and arterial stiffness [25]. However, inclusion of these factors is more likely to improve upon, rather than detract from, our observed findings.

We report missing/erroneous values for 110 participants (5.8 %). However, we do not feel that this number reflects the true error rate with the device but is a reflection of our protocol design. Participants were approached in a community setting (shopping centre) and assessed in that same setting within 10-minutes of volunteering for the study. As a result, we believe that for some patients the scenario in which they were placed may have resulted in a raised BP and produced an erroneous diastolic BP of over 120 mmHg. This occurred in 29 participants or 1.5 % of the sample. Missing readings for cBP and AIx were reported in a further 81 participants (4.3 %). These reflected an inability to record a suitable quality pressure wave on the first attempt and where a second attempt was not possible (i.e. due to time constraints, participant unwillingness). In practice, however, a repeat attempt is likely to be possible and therefore the missing readings observed here are unlikely to be as high in other settings (e.g. clinical practice). For reasons described we performed only one peripheral BP assessment prior to pulse wave assessments. In clinical practice it is recommended the average of two measures is used to determine office BP [27]. In our study, however, we are aggregating values across groups and assessing distribution of mean values as opposed to assessing individual values for clinical decisions. Any inaccuracies in individual values are likely to be negated by regression toward the mean. Likewise, in clinical practice where individual data are required, two peripheral BP assessments can be performed in accordance with current guidelines.

Normotensive individuals were identified based on their peripheral BP as measured within the study and using the systolic BP determined from the oscillometric device. Thus, it is likely that a number of individuals presenting with a BP > 140 mmHg could have had artificially inflated values (white coat hypertension). It is also possible that a number of individuals with hypertension would have been missed (masked hypertension). The fact that only one peripheral BP assessment per participant was performed may also further confound these two factors. This may therefore present a markedly different population to the “normotensive” cohort of McEniery et al, in which all participants underwent a clinical diagnosis of hypertension.

## Conclusions

We present for the first-time non-invasive estimation of central blood pressure and arterial stiffness parameters from a commercially available, validated automated oscillometric device in a large community-based population and under deliberately robust settings. Measures of central blood pressure and pressure wave augmentation comply with known age and sex associations previously observed from reference non-invasive techniques. Estimates from more controlled settings (e.g. primary care) and with additional demographic parameters is warranted, particular to identify those factors influencing central haemodynamic and arterial stiffness parameters. Whilst the utility of arterial assessment at the primary care level becomes realised, this must be accompanied by concomitant clinician knowledge and understanding of their meaning and clinical significance.

## Competing interests

SW and CCM are inventors of a patent which is partly used in ARCSolver. There are no other competing of interest.

## Authors’ contributions

DN constructed the concept of the study, performed the analysis, interpreted the data, drafted the manuscript and approved the final manuscript to be published. SW and BH performed data collection, participated in data analysis and interpretation of data, revised the manuscript for important intellectual content and approved the final manuscript to be published. DL, SF, AW and CH participated in data analysis and interpretation of data, revised the manuscript for important intellectual content and approved the final manuscript to be published. All authors’ read and approved the final manuscript.

## Author’s information

This work and its publication was partly supported by a grant of the government of Lower Austria and EFRE, contract number WST3-T-81/015-2008.

## Pre-publication history

The pre-publication history for this paper can be accessed here:

http://www.biomedcentral.com/1471-2261/12/48/prepub
